# Vitamin D and Probability of Developmental Disorders among Perinatally HIV-Affected and Unaffected Ugandan Children

**DOI:** 10.3390/nu15092020

**Published:** 2023-04-22

**Authors:** Jorem E. Awadu, Bruno Giordani, Alla Sikorskii, Catherine Abbo, Jenifer I. Fenton, Sarah Zalwango, Amara Esther Ezeamama

**Affiliations:** 1 Department of Psychiatry, Michigan State University, East Lansing, MI 48823, USA; awadujor@msu.edu (J.E.A.);; 2 Department of Psychiatry, University of Michigan, Ann Arbor, MI 48109, USA; 3 Department of Psychiatry, Makerere College of Health Sciences, Kampala 00256, Uganda; 4 Department of Food Science and Human Nutrition, Michigan State University, East Lansing, MI 48823, USA; 5 Directorate of Public Health and Environment, Kampala Capital City Authority, Kampala 00256, Uganda

**Keywords:** vitamin D deficiency, vitamin D, 25-hydroxyvitamin D, developmental disorders, ASD, ADHD, resiliency, functional impairment

## Abstract

We tested the hypothesis that vitamin D deficiency (VDD) is associated with higher developmental disorder probability in 604 children with perinatal HIV infection (CPHIV, *n* = 199), HIV exposed and uninfected (CHEU, *n* = 196), and HIV unexposed uninfected (CHUU, *n* = 201). Children at 6–18 years old and their adult caregivers were assessed at enrollment, 6, and 12-month follow-ups. Serum 25-hydroxyvitamin-D (25OHD) levels in children quantified per the NHANES protocol were used to define VD categories as VDD (25OHD < 20 ng/mL), VD insufficient (VDI, 20 ≤ 25OHD ≤ 25 ng/mL), and VD sufficient (VDS = reference group if 25OHD > 25 ng/mL). Perinatal HIV status per DNA polymerase chain reaction/HIV rapid diagnostic tests included: CPHIV, CHEU, and CHUU. Developmental stage was defined as pre-adolescent (age < 11) vs. adolescent (age ≥ 11) years. Caregiver responses to standardized questions from Behavioral Assessment System for Children, Third Edition (BASC-3), were used to calculate probability scores for four disorders, namely: autism (ASD), attention deficit & hyperactivity (ADHD), emotional behavioral disorder (EBD), functional impairment (FI), and resiliency at 0, 6 and 12 months. Multivariable longitudinal models estimated VD-associated standardized mean difference (SMD) and corresponding 95% confidence intervals (95% CI) in respective probability scores in Statistical Analysis Software (v.9.4). Baseline VDD vs. VDS predicted higher probability scores of moderate clinical importance for ASD, ADHD, EBD, and higher FI among pre-adolescents (SMD = 0.32 to 0.40, 95% CI: 0.00 to 0.74). VDD was not associated with resiliency or any developmental disorders among adolescents. VDD predicted higher developmental disorder and FI scores over 12 months in a developmental stage-dependent manner. This relationship requires further understanding to appropriately target future interventions.

## 1. Introduction

Vitamin D deficiency (VDD) is common in the general population [1,2], though the prevalence is higher in people living with HIV (PLWH) [3,4,5,6,7,8,9,10,11,12,13,14,15]. An estimated 90% of serum vitamin D is derived from the ultraviolet B radiation from the sun; the remaining 10% arises from dietary sources such as meat, oily fish, eggs, and dairy products [2,16]. Vitamin D’s critical role in skeletal homeostasis through its impact on calcium, phosphate, and magnesium metabolism, which promote proper bone mineralization and linear growth [17,18], is well established. Emerging evidence shows that beyond the skeletal system, vitamin D affects several other organ systems through the genomic action of its biologically active form—i.e., 1,25-(OH)_2_D, on the vitamin D receptor (VDR) [19]. VDR and 25-hydroxyvitamin D (i.e., the hydroxylated form of VD produced by the liver) are found in the enteric and central nervous systems, including the brain [20,21]. This observation suggests that vitamin D may also be important for immune homeostasis and brain development, maturation, and function. Particularly relevant for neurodevelopment is vitamin D’s function as a neuro-steroid hormone with receptors in brain structures such as the cerebellum, hypothalamus, basal ganglia, thalamus, and hippocampus [20,22]. The expression of VDR in the hippocampus and the limbic system implicates vitamin D in basic emotions and drives, learning, memory, neurogenesis, and brain plasticity [23,24,25]. For example, VDD has been associated with neuroinflammation [26], interference with neurotransmission within the central and enteric nervous systems [27], higher prevalence of developmental disorders such as autism spectrum disorder (ASD) [28,29], attention deficit and hyperactivity disorder (ADHD) [30,31,32,33], poor socio-emotional adjustment [34], lower resiliency [35], and higher functional impairment [36,37]. Of note, these developmental disorders tend to co-occur with dysphoria, depression, and central nervous system disorders such as epilepsy and adversely affect functional survival, social, educational, and developmental trajectories throughout the life course [31].

Developmental disorder prevalence has increased over the past 40 years, with global ASD prevalence rising from 1in 5000 in 1975 to 1in 45 in 2014 [38,39]—a prevalence rate largely applicable to high-income countries. The extent to which this 110-time increase represents an organic increase in the number of children impacted by ASD and/or represents a change in diagnostic patterns and wider recognition is unknown. Nevertheless, developmental disorders represent a public health challenge with few known modifiable targets. Hence, the need to understand the different etiological factors that contribute to a range of developmental disorders is a high public health priority to uncover potentially modifiable determinants and inform health system planning, including resource expenditure on rehabilitation services.

VDD is a potentially modifiable determinant, but its role in the etiology of developmental disorders in children from resource-constrained settings is unknown. An understanding of the relationship of VDD to developmental disorders is especially valuable among children with comorbid chronic diseases such as perinatally acquired HIV infection, where the prevalence of VDD is even higher due to the impact of the HIV virus and HIV-related treatment. For example, among children with perinatally acquired HIV infection (CPHIV) being treated with protease inhibitors (PI) or non-nucleoside reverse transcriptase inhibitors (NNRTI), current antiretroviral therapy (cART) use has been linked with conversion of vitamin D metabolites to its non-biologically active form—i.e., calcitroic acid [40] predisposing such populations to functional VDD. Recently published work by our team in a sample of Ugandan children with and without perinatal HIV infection/ART exposure [41] provided reassuring evidence that developmental disorder probability scores in late childhood and adolescent years of life were similar for children with perinatally acquired HIV (CPHIV) and children who are HIV-exposed but uninfected compared to children HIV unexposed uninfected (CHUU) [41]. However, peripartum exposure to certain ART regimens—specifically, sdNVP + AZT + 3TC, was associated with elevated probability scores for ADHD, ASD, emotional behavioral disorder (EBD), and functional impairment (FI) in both CHEU and CPHIV groups compared to CHUU.

Here we extend the scope of available information on developmental disorder prevalence in an understudied, highly vulnerable population of HIV-impacted children by investigating whether baseline VDD is associated with higher developmental disorders risk after adjusting for caregiver sociodemographic, mental health, behavioral and lifestyle factors. We have further explored whether this association was affected by developmental stage, perinatal HIV status, and peripartum ART exposure. We hypothesize that VDD status will be associated with elevated developmental disorder probability in the potentially developmental stage, HIV status, and peripartum ART exposure status dependent manner. Results from this study will provide an important rationale for determining the possible utility of vitamin D supplementation for improving the neurodevelopmental trajectory of vulnerable children with and without HIV infection and concomitant cART exposure.

## 2. Materials and Methods

### 2.1. Study Participants

Children and their primary caregivers (i.e., 6–18 and ≥18 years of age, respectively) were recruited into two prospective cohort studies and followed up between 16 March 2017, and 30 June 2021. By design, three groups of children (i.e., perinatally HIV-infected [CPHIV], HIV-exposed uninfected [CHEU], and HIV-unexposed uninfected [CHUU]) together with their caregivers were recruited. In total, we recruited 750 children (*n* = 250 for each of the three groups, [i.e., CPHIV, CHEU, and CHUU]). Perinatally, HIV-infected children were recruited from a hospital in which they received routine care in urban Kampala, Uganda. CHEU were identified from the same hospital using records abstracted from their Early Infant Diagnosis (EID) registers, while CHUU were recruited following referrals by child-caregiver dyads already enrolled in our study. Rapid HIV diagnosis tests were utilized in ascertaining the current HIV status of both CHEU and CHUU at enrolment. We followed each child-caregiver pair for a year or up until they were lost to follow-up. Study measures were administered at baseline, six, and twelve months.

### 2.2. Eligibility/Exclusion Criteria

Eligible study participants had to be 6–18 (children) and ≥18 years old (parents) at enrolment. A primary caregiver was defined as a person who had cared for the target child for at least six months by study enrolment. Only children who were born in a hospital were enrolled because their ART exposure status in utero, HIV status for both the mother and child, and the mother’s participation in the PMTCT program were verifiable. Details on maternal ART regimen, target child’s birth weight, and APGAR scores were obtained from hospital delivery records. Children not born in a hospital setting and caregiver-child pairs whose birth, care, and antenatal information could not be confirmed were ineligible as it was impossible for their HIV status and ART exposures in pregnancy to be verified. For these secondary analyses, a lack of availability of information for VD and developmental disorders were additional exclusion criteria.

### 2.3. Ethical Approval

The different study protocols were reviewed and approved by members of the research ethics boards of Michigan State University (IRB Protocol numbers: 16-828 and 205), Makerere University College of Health Sciences, School of Medicine (Protocol REC REF numbers: 2017-017 and 2018-099), and the Uganda National Council for Science and Technology (Protocol #s: SS4378 and HS 2466). Study caregivers and children were required to provide informed consent and, where applicable, assent before participating in the study.

### 2.4. Definitions of Outcomes: Developmental Disorder, Functional Impairment, and Resiliency

The likelihood for ASD, ADHD, EBD, functional impairment, and resiliency indices was defined per proxy—i.e., responses from primary caregivers to items in the Behavioral Assessment System for Children, Third Edition (BASC-3) manual [42]. Manufacturer instructions for scoring and summing individual items were followed in four separate disorder probability indices, an index of functional impairment, and an index of resiliency as continuous outcome variables (z-scores) compared to age-matched Ugandan CHUU. Questionnaire items were first adapted to fit Uganda’s cultural context and then forward and back-translated as we already described [23]. All participants received snacks or refreshments before being assessed to minimize distraction from feeling hungry/tired on responses.

Autism Spectrum Disorder (ASD) Probability Index: Derived from caregiver response to 13 or 18 BASC-3 questionnaire items (for children <11 or ≥11 years, respectively) where they verbally evaluated their children’s behavior, communication, and social skills. A high ASD probability index score is positively associated with ratings of behavior in children with a clinically diagnosed autism spectrum disorder and indicative of an increased likelihood to display unusual behaviors, including but not limited to problems with the development/maintenance of peer relationships [42,43].

Attention Deficit or Hyperactivity Disorder (ADHD) Probability Index: Index derived from the summing of 11 or 9 questionnaire items (if child <11 or ≥11 years, respectively) that are positively associated with behavioral ratings of children clinically diagnosed with ADHD. High scores on this index are indicative of difficulty in school or tasks requiring sustained focus because of the inability to maintain attention, plan, make decisions, or moderate activity intensity levels [43].

Emotional Behavioral Disorder (EBD) Probability Index: Derived by summing 30 or 20 items (i.e., if child <11 and ≥11 years, respectively). Higher scores on this index are associated with behavioral scores indicative of children with emotional/behavioral disturbance or disability within school settings. The scores are also suggestive of an increased likelihood of disruptive, untypical, and antisocial behaviors or high levels of anger, sadness, and pessimism—which strain social relationships both in peers and adults [43].

Functional Impairment (FI): This index was derived by the summation of 44 items capturing how difficult it is for a child to successfully engage in appropriate behaviors when interacting with peers, performing age-appropriate tasks, mood regulation, and accomplishing of school activities. A higher score on this index suggests difficulty in responding appropriately to everyday social interactions in varied settings [43].

Resiliency Index: This index is derived from nine BASC-3 items assessing how one adapts their behaviors to cope with change, setback recovery, and resolution of problems in adversity. High scores on this index are indicative of a high likelihood of being resilient in the short term and are associated with good mental health [43].

### 2.5. Primary Determinant: Vitamin D (25-Hydroxyvitamin)

25-Hydroxyvitamin (herein referred to as vitamin D) was measured using serum fractions separated from blood samples extracted from study participants and stored at −80 °C. The stored samples were then transported to Sparrow Laboratories (East Lansing, Michigan) for vitamin D levels measured using the high-performance liquid chromatography (HPLC MS/MS) method following the NHANES protocol described prior [34]. Low (referred to as vitamin D deficient [VDD], medium (i.e., vitamin D insufficient [VDI]), and high vitamin D (vitamin D sufficient [VDS]) status was defined following 25-hydroxyvitamin levels corresponding to <20 ng/mL, 20–25 ng/mL, and >25 ng/mL, respectively.

### 2.6. Other Measures

Sociodemographic Factors: A child’s biological sex was defined as male vs. female; their age and education level accomplished (in years) and developmental stage as pre-adolescent vs. adolescent (i.e., <11 vs. ≥11 years, respectively). The same applied to caregiver sex (male vs. female), chronological age and education (in years), as well as social standing, stress, anxiety, depression, and social support, which were defined as low vs. high.

Caregiving Context: We used the perceived stress scale to measure the caregiver’s acute psychosocial stress levels over a month prior to the time of assessment. The scale scores ranged from 0 (indicative of no stress) to 40 (indicative of the highest possible stress level) [24,25]. The stressful life events questionnaire was used to assess lifetime stress (i.e., the sum of occurrences for 13 adverse experiences over the life course [26]. Caregiver rating of their personal or subjective social standing (scores ranging from 1 to 10 [lowest to highest rating], respectively) was derived using the MacArthur scale of subjective social standing [27]. An adapted version of the Barkin index of maternal functioning scale (with scores ranging from 1 to 120 [lowest to highest], respectively) [28,29] was utilized to assess functioning in the caregiving role. Depressive and anxiety symptoms in caregivers were measured using 15 and 10 items, respectively, from the Hopkins Symptom Checklist-25 [30,31]. Similar to our previous studies, social support was measured using a summed score of eight questions where caregivers agreed or disagreed with statements pertaining to the ease of access to desirable financial, emotional, and physical support resources [32].

### 2.7. Statistical Analyses

The summarized relationships between vitamin D (VD) status (i.e., at baseline) and time-averaged developmental disorder likelihood, resiliency, and functional impairment indices were derived using analysis of variance and chi-square tests (for analysis of continuous and categorical variables, respectively). Multivariable linear mixed-effects models were implemented to quantify VD-related differences in developmental disorder outcome measures over 12-month follow-ups using SAS PROC MIXED. In all multivariable models, confounders such as caregivers’ age, sex, education, anxiety, social support, social standing, adverse lived experiences, socioeconomic status, depression, and stress were adjusted based on subject matter knowledge. The random effect of the caregiver was included in all models to account for the nesting of children within households. Time was entered as a class variable to model and potentially identify any non-linear patterns in disorder outcomes, and an interaction between time and VD categories was examined to assess potential variation in VD association with respective outcome measures over within observation periods. In the absence of VD by time interaction, time-averaged associations between VD and repeated assessments of respective disorders over 12 months were presented. Since outcomes were standardized by age and sex, differences in the least square means from the mixed models were an estimate of standardized mean differences (SMD)—a measure comparable to Cohen’s effect size, thus permitting the determination of clinical importance for VD-associated differences in developmental disorders. Per prior precedent based on studies on quality of life, |SMD| of ≥0.33 were deemed clinically important for developmental disorder outcomes because of their wide-ranging lifelong implications in affected populations [33]. Hence, for interpretation, |SMD| < 0.33, 0.33 ≤ |SMD| < 0.50, and |SMD| ≥ 0.50 are considered of small to modest, moderate, and large clinical importance, respectively.

To explore developmental stage, perinatal HIV status, and peripartum ART exposure type as potential moderators of VD-associated variations in respective outcomes over 12 months, VD by each factor interaction was introduced in multivariable models. For this exploratory analysis, potential heterogeneity was indicated by the *p*-value for the interaction term <0.1. In that case, results for VD-related SMD in developmental disorders were presented separately for each stratum of a potential modifier. In the absence of heterogeneity, results are presented for the overall sample. All analyses were performed with SAS version 9.4 (SAS Institute, Inc., Cary, NC, USA). All hypothesis tests were two-sided at alpha = 0.05.

Given the sample sizes of VD deficient (*n* = 205), insufficient (*n* = 294), and sufficient (105) children, a medium effect size of f-squared of 0.25 was detectable with a power of 0.80 at 0.05 level of significance in the ANOVA comparing three groups on probability scores at each time point. In the pairwise comparisons of VD groups, the detectable effect sizes (Cohen’s d) were 0.34 (for VDD versus sufficient), 0.32 (insufficient versus sufficient), and 0.26 (insufficient versus deficient) at each time point. Smaller effect sizes for between-group differences were detectable in the longitudinal analysis due to the reduction of error variance with three repeated measures.

## 3. Results

Of 604 eligible study participants with information on VD and developmental disorders, 205, 294, and 105 were vitamin D deficient (VDD), vitamin D insufficient (VDI), and vitamin D sufficient (VDS), respectively. At baseline, vitamin D levels varied significantly (*p* ≤ 0.01) for child age, with those who were VDS being older (M = 11.97, SD = 3.88) followed by VDI (M = 11.70, SD = 3.54) and VDD at (M = 11.33, SD = 3.65). However, children in the D status categories were not different according to sex, perinatal HIV status, as well as Apgar and birth weight indices. Caregivers were similar for all factors (i.e., age, education, social standing, recent life stress, anxiety, social support, and depression). Similarly, the likelihood for developmental disorders (i.e., ASD, ADHD, DSD, and EBD), as well as resiliency and functional impairment among study children, was similar for children across the different VD categories (Table 1).

### 3.1. Developmental Disorder Likelihood by HIV Status across 12 Months

Regardless of developmental stage, the likelihood of developmental disorders, resiliency, and functioning indices improved significantly across the study period. The likelihood for all disorders (except for ADHD and resiliency in pre-adolescents and EBD among adolescents) improved significantly across one year. (Table 2). Regardless of HIV status, the reduction in the likelihood of DSD and functional impairment was moderately lower but significant by one year (all *p* < 0.05) for all children. There was a significant reduction in the likelihood of ASD, DSD, and FI among CPHIV (all *p* < 0.05). Statistically significant reduction in likelihood for all developmental disorders (except for EBD), increased resiliency, and functioning was observed among both CHEU and CHUU (all *p* < 0.05) (Table A1).

### 3.2. Vitamin D Relationship to Developmental Disorder Likelihood by Developmental Stage

With adjustment for HIV status and time, there was a statistically significant increased likelihood for ASD, ADHD, and EBD of moderate magnitude (M = 0.34–0.38, SD = 0.02–0.70) among VDD compared to VDS pre-adolescents. Likewise, VDD pre-adolescents had a moderately higher likelihood of functional impairment compared to their VDS counterparts, although the relationship was non-statistically significant. There was no difference among VDI and VDS pre-adolescents for developmental disorder likelihood, resiliency, and functioning indices. In adolescents, there was no difference in developmental disorder probability risk, resiliency, or functional impairment by vitamin D status (Table 3).

### 3.3. Vitamin D Relationship to Developmental Disorder Likelihood in Pre-Adolescents (Adjusted Models)

With adjustment for HIV status and time, there was a statistical sign when caregiver factors (i.e., education, age, anxiety, sex, social support, social standing, recent life stress, adverse life events, and depression) were adjusted for in multivariable model 2, the likelihood for ASD (SMD = 0.32, 95% CI: 0.00, 0.64), ADHD (SMD = 0.38, 95% CI: 0.07, 0.70), and EBD (SMD = 0.40, 95% CI: 0.05, 0.74) and FI (SMD = 0.35, 95% CI: 0.02, 0.68) remained moderately statistically significant for VDD compared to VDS pre-adolescents. The likelihood for developmental disorders (i.e., ASD, ADHD, and EBD) and functional impairment in VDD compared to VDS pre-adolescents stayed moderately statistically higher when Apgar scores and birth weight (child factors) together with parental factors were adjusted for in the multivariable model 3 (Table 4).

### 3.4. Child and Maternal Factors Relationship to Developmental Disorders, Resiliency, and Functional Impairment Indices

Per unit (kilogram) increase in birthweight was associated with a small but significant reduction in ADHD probability scores (SMD = −0.19, 95% CI: −0.34, −0.03). Likewise, per unit increase in child Apgar score was associated with a small but significant reduction in ASD (SMD = −0.11, 95% CI: −0.19, −0.04), DSD (SMD = −0.11, 95% CI: −0.19, −0.03), FI (SMD = −0.13, 95% CI: −0.21, −0.05) and increased resiliency (SMD = 0.11, 95% CI: 0.03, 0.18). Caregivers who reported lower vs. higher incidence of life adversity were more likely to rate their pre-adolescent children as having ASD (SMD = −0.23 to −0.53, 95% CI: −0.73, −0.04), ADHD (SMD = −0.35 to −0.49, 95% CI: −0.70, −0.14), EBD (SMD = −0.24 to −0.67, 95% CI: −0.90, −0.03), DSD, and functional impairment (SMD = −0.21 to −0.66, 95% CI: −0.88, −0.01) (Table A2). There was no significant interaction effect for vitamin D by early ART and vitamin D by HIV status, with the lowest correlation of all outcomes being above 0.45 and 0.35 for the former and latter, respectively.

## 4. Discussion

In this sample of Ugandan children, the prevalence of VDI and VDD were high, and VDD was associated with worse functional impairment, lower resiliency, and higher developmental probability scores—specifically ASD, ADHD, and EBD among pre-adolescents over 12 months follow-up. The high prevalence of VDI/VDD may be suggestive of the need to clinically monitor vitamin D status in this population, given VD’s importance in multiple organ systems and clinical outcomes, including brain health [17,18,19,20,21,22]. Hence, routine clinical assessments could be instrumental to health promotion in this population in two ways. First, this information will facilitate the identification of sub-populations at high risk of a range of adverse clinical and neurodevelopmental trajectories for nutrition counseling and potential interventions when indicated. Secondly, knowledge of VD status may encourage behaviors that enhance dietary quality in the population with a range of health benefits of potential relevance for optimizing brain health [44].

In line with our hypothesis, VDD was associated with higher developmental disorders probability scores, but this association was developmental stage-dependent and constrained to pre-adolescents. Specifically, we observed that compared to VDS pre-adolescents, VDD peers had elevated ASD, ADHD, and EBD as well as functional impairment probability scores over 12 months follow-up. Our findings are consistent with previous studies associating VDD in children with increased risk of ASD [1,28,29,45,46] and ADHD [30,31,32,33]. A multinational study of VD supplementation vs. placebo among adults from the United States and Norway found that the VD-supplemented group demonstrated greater stress resiliency [36] and neuropsychological performance over the cold winter months compared to the placebo group. Further support for our findings and study hypothesis comes from a large cross-sectional study in Germany that found VD-sufficient adults were more resilient compared to their VDI/VDD peers [37]. Together, these epidemiologic studies and available information implicating VD levels in immune function and CNS homeostasis [26,27] suggest that improving VD status in vulnerable populations may translate to cognitive benefits and potentially reduce the onset and/or severity of developmental disorder symptoms in vulnerable populations. Of note, VDD/VDI prevalence is high in both resource-rich and LMIC settings, and this risk is modifiable via supplementation in vulnerable populations where the benefit from supplementation will be maximized and intervention implemented at a relatively low cost. Unfortunately, to date, demonstrated health benefits of VDD supplementation in various contexts have been variable in part due to a limited understanding of applicable mechanisms and the population sub-groups at high risk of VDD-associated adverse clinical and developmental outcomes [1,47,48,49]. Hence future studies that identify sub-groups at high risk of VDD-related adverse neurodevelopmental trajectory will be important to confirm our findings. Further, mechanistic studies that elucidate applicable and potentially modifiable biological pathways by which VDD-impacts cognition will be important to inform intervention strategies, where warranted.

In this sample, VDD was most consistently associated with higher disorder probability scores and functional impairment among pre-adolescent children, with limited evidence associating VDD and respective outcomes among adolescents. This developmental stage-dependent association was surprising to us; the reason for variation in VD association with outcomes by developmental stage is unclear and requires further elucidation. Our findings among pre-adolescent children align with our prior report of more serious socio-emotional adjustment problems in VDD vs. VDS pre-adolescent Ugandan children that were between 6 and 10 years old [34]. Unlike our study, a nationwide health interview and examination survey of 9068 German children and adolescents aged 3–17.9 years found higher vitamin D levels to be associated with lower problems in peer relationships and emotional symptoms without any variation by developmental stage [35]. The results among German children provide partial support for observations noted herein, but differences in contextual factors across countries, including variations in VD fortification practices and availability/affordability of VD-rich foods such as fish, milk, eggs, and animal protein, may partially explain the differences in VD association across studies. Given the sub-group-specific finding in this sample, it is also possible that the difference in association by developmental stage is behaviorally mediated. For example, we cannot exclude the possibility that a greater capacity to engage in masking behaviors [50] reduced the likelihood of caregivers observing/reporting behaviors suggestive of milder forms of developmental disorders. Further caregiver-related behavioral, environmental, or contextual factors may partly account for disparate associations by child developmental stage to unknown degrees. Specifically, caregivers may have more control over the dietary intake and activity patterns of younger children in ways relevant to their VD status. Similarly, caregivers’ capacity to observe and monitor behaviors may be higher for younger compared to older children. The observation that vitamin D relationship to outcomes only became statistically significant after adjustment for caregiver factors like anxiety, depression, and stress as well as child Apgar scores highlight the importance of caregiver and child contextual factors as potential drivers of the association between VDD, developmental disorders, resiliency, and functional outcomes among pre-adolescents. Hence, future studies in LMIC settings are needed to clarify the observation of potential variation in these relationships according to developmental stages in this study and the potential contribution of modifiable environmental and/or contextual risk factors.

We have previously reported that exposure to suboptimal ART was independently associated with higher developmental disorder probability scores [41] and thus specifically investigated HIV and peripartum ART exposure type as potential mediators/modifiers of VD association with respective outcomes. We found no evidence that the observed relationship between VD, developmental disorders, resiliency, and functional impairment was mediated or modified by peripartum HIV status or peripartum ART exposure type. This suggests that the VDD-associated risk of outcomes in this study is similar for CPHIV, CHEU, and CHUU subgroups and does not vary according to peripartum ART history among CPHIV and CHEU. In other words, our findings suggest that future VD supplementation efforts may equally benefit all pre-adolescent children regardless of peripartum HIV status or peripartum ART exposure type. Further research to understand the mechanisms by which VD status relates to developmental disorders, resiliency, and functional impairment among pre-adolescents and adolescents is needed. VDD has, for example, been associated with gut dysbiosis, which creates a pathway for developmental disorders resulting from a leaky blood-brain barrier. VD has also been implicated in brain development, maturation, and functioning through its impact on the release of neurotransmitters, such as serotonin and dopamine and others, which have a direct impact on human behavior expression. In addition, more research on the different VD canonical forms of a mechanistic pathway to developmental disorders is needed for the furtherance of our understanding.

### Limitations, Strengths, and Future Directions

We are unable to exclude residual confounding as an alternate explanation for VD association with respective outcomes due to the observational nature of this study. In addition, although BASC-3, as used in this study, aligns with the Diagnostic and Statistical Manual of Mental Disorders (DSM-5) in the identification of mental and neurodevelopmental disorders, the outcomes as defined do not directly correspond to clinical diagnosis. The accuracy of diagnosing ASD, ADHD, and EBD outcomes could be enhanced by consensus clinical diagnosis and/or the use of alternative gold-standard questionnaire-based or observational behavior assessments such as the Autism Diagnostic Observation Schedule (ADOS) [51] or Autism Diagnostic Interview-revised (ADI-R) [52] for consensus diagnosis of developmental disorders will further help our understanding of these relationships. Despite these limitations, our study has the following strengths that should enhance confidence in our findings. First, this study’s longitudinal study design provides inference regarding temporal sequence in the relationship of VD to respective disorders and thus enhances inferential rigor in the current body of work that is mostly cross-sectional in nature [47]. Additional strengths of the present study include rigorous statistical analyses with appropriate control for potential lack of independence within participants and households as well as analytic control for a wide range of potential confounders resulting in the most de-confounded association possible within the constraints of our observational study design.

Considering our unexpected finding that VDD is associated with higher developmental disorders probability scores in a developmentally stage-dependent manner, additional studies to confirm or refute this observation are warranted. In addition, studies examining the mechanistic pathway underlying VDD and developmental disorder association, as well as any variations in relation to outcomes according to how VD is measured, whether total serum D2/D3 and/or bio-available metabolites of VD will be instrumental to designing future interventions. Lastly, future investigations will be enhanced by the inclusion of clinically diagnosed developmental disorder outcomes as complementary measures to the outcomes defined per standardized questionnaires in this study.

## 5. Conclusions

In this sample, VDD status was associated with higher developmental disorder and functional impairment probability scores over 12 months among pre-adolescent but not among adolescents. Reasons for variation in relation to developmental stage are unclear and specific investigations to understand this variation may inform the targeting of nutritional interventions in vulnerable populations with possible benefits for improving developmental trajectory, functioning, and quality of life in vulnerable populations.

## Figures and Tables

**Table 1:** Baseline description of study base with respect to vitamin D deficiency, insufficiency, or sufficiency status.

	Overall (*n* = 604)	VDD (*n* = 205)	VDI (*n* = 294)	VDS (*n* = 105)	Unadjusted Comparison ANOVA/χ^2^
	*n* (%)	*n* (%)	*n* (%)	*n* (%)	
**Child Factors**					
% Female	316 (52.2)	110 (53.9)	152 (51.4)	54 (51.4)	0.84
Perinatal HIV Status					
Infected	199 (33.4)	66 (33.17)	89 (44.72)	44 (22.11)	0.06
Exposed uninfected	196 (32.9)	66 (33.67)	102 (52.04)	28 (14.29)	
Unexposed uninfected	201 (33.7)	72 (35.82)	101 (50.25)	28 (13.93)	
	**Mean** (**SD**)	**Mean** (**SD**)	**Mean** (**SD**)	**Mean** (**SD**)	
Age (years)	11.33 (3.65)	10.49 (3.54)	11.7 (3.54)	11.97 (3.88)	<0.001
Apgar	8.3 (0.96)	8.39 (0.99)	8.28 (0.95)	8.26 (0.91)	0.15
Birth Weight (Kgs)	3.3 (0.52)	3.33 (0.56)	3.25 (0.50)	3.22 (0.50)	0.22
**Caregiver Factors**					
Age (years)	37.8 (11.25)	36.56 (11.21)	38.4 (11.28)	38.62 (11.19)	0.20
Education (years)	6.58 (3.75)	6.95 (3.72)	6.38 (3.55)	6.4 (4.28)	0.35
Social Standing McArthur Scale	3.4 (1.44)	3.44 (1.55)	3.41 (1.42)	3.33 (1.27)	0.89
Recent Life Stress (RLS)	7.95 (3.72)	7.88 (3.59)	8 (3.84)	8 (3.72)	0.96
Anxiety Score	7.78 (6.56)	8.25 (6.39)	7.71 (6.63)	7.08 (6.73)	0.26
Social support Score	11.59 (5.05)	12.1 (4.56)	11.14 (5.25)	11.76 (5.38)	0.17
Depressed	10.49 (8.36)	10.8 (7.91)	10.39 (8.68)	10.17 (8.44)	0.59
**Child Disorder, Functional Impairment, and Resiliency Outcomes**					
Autism Spectrum Disorder (ASD)	0 (0.99)	0.07 (0.99)	−0.03 (0.98)	−0.06 (1.01)	0.67
Attention Deficit and Hyperactivity Disorder (ADHD)	−0.04 (1.03)	−0.05 (1.03)	−0.06 (1.06)	0.02 (1.0)	0.45
Developmental Social Disorder (DSD)	−0.06 (0.99)	−0.01 (0.96)	−0.07 (0.99)	−0.13 (1.04)	0.45
Emotional Behavior Disorder (EBD)	−0.02 (0.98)	0.04 (0.97)	−0.07 (0.96)	−0.03 (1.06)	0.30
Resiliency Index (RI)	0.07 (1.04)	0.09 (1.04)	0.05 (1.03)	0.09 (1.05)	0.91
Functional Impairment Index (FII)	−0.01 (1.02)	−0.01 (0.99)	−0.02 (1.04)	0.02 (1.06)	0.58

**Table 2:** Child disorder, functional impairment, and resiliency outcomes by developmental stage across 12 months.

Outcomes	Baseline (*n* = 605)	Month 6 (*n* = 597)	Month 12 (*n* = 444)	*p*-Trend
	Mean (SD)	Mean (SD)	Mean (SD)	
**Overall** (**Regardless of Developmental Stage**)				
Autism Spectrum Disorder	−0.00 (0.98)	−0.15 (0.97)	−0.24 (0.93)	<0.0001
Attention Deficit Hyperactivity Disorder	−0.04 (1.03)	−0.17 (1.00)	−0.26 (1.03)	0.0001
Developmental Social Disorder	−0.06 (0.98)	−0.24 (0.95)	−0.33 (0.89)	<0.001
Emotional Behavior Disorder	0.02 (0.97)	−0.09 (1.03)	−0.15 (0.99)	0.03
Resiliency	0.07 (1.01)	0.22 (0.94)	0.23 (0.90)	<0.0001
Functional Impairment Index	−0.01 (1.01)	−0.15 (1.05)	−0.31 (0.96)	<0.0001
**Pre-Adolescents**	(***n* = 247**)	(***n* = 244**)	(***n* = 247**)	
	**Mean** (**SD**)	**Mean** (**SD**)	**Mean** (**SD**)	
Autism Spectrum Disorder	−0.03 (0.96)	−0.13 (0.99)	−0.28 (0.94)	<0.001
Attention Deficit Hyperactivity Disorder	−0.12 (1.05)	−0.13 (1.09)	−0.29 (1.08)	0.12
Developmental Social Disorder	−0.08 (0.99)	−0.26 (0.97)	−0.39 (0.89)	<0.0001
Emotional Behavior Disorder	0.00 (1.02)	−0.04 (1.12)	−0.20 (1.02)	0.04
Resiliency Index	0.13 (0.93)	0.24 (0.83)	0.21 (0.79)	0.05
Functional Impairment Index	−0.07 (1.02)	−0.20 (1.02)	−0.38 (0.93)	<0.001
**Adolescents**	(***n* = 358**)	(***n* = 353**)	(***n* = 197**)	
	**Mean** (**SD**)	**Mean** (**SD**)	**Mean** (**SD**)	
Autism Spectrum Disorder	0.020 (1.01)	−0.17 (0.97)	−0.20 (0.94)	<0.001
Attention Deficit Hyperactivity Disorder	0.01 (1.02)	−0.17 (0.97)	−0.23 (0.94)	<0.001
Developmental Social Disorder	−0.05 (0.98)	−0.22 (0.93)	−0.25 (0.92)	0.002
Emotional Behavior Disorder	−0.04 (0.95)	−0.12 (0.95)	−0.09 (0.96)	0.35
Resiliency Index	0.03 (1.10)	0.22 (0.99)	0.25 (1.07)	0.003
Functional Impairment Index	0.03 (1.03)	−0.12 (1.03)	−0.15 (1.03)	0.02

**Table 3:** Vitamin D in relationship to developmental disorder probability indices among pre-adolescents and adolescent Ugandan children.

		Pre-Adolescents (*n* = 246)		Adolescents (*n* = 358)		χ^2^
	VD Groups	LSM (SE)	SMD (95% CI)	LSM (SE)	SMD (95% CI)	VD * Adolescent Interaction
**Autism Spectrum Disorder**	VDD	−0.06 (0.08)	0.34 (0.02, 0.66)	−0.09 (0.08)	−0.01 (−0.26, 0.25)	0.25
	VDI	−0.19 (0.08)	0.21 (−0.11, 0.52)	−0.15 (0.07)	−0.07 (−0.30, 0.17)	
	VDS	−0.40 (0.14)	**Ref**	−0.09 (0.10)	Ref	
**Attention Deficit and Hyperactivity Disorder**	VDD	−0.12 (0.08)	0.34 (0.03, 0.64)	−0.12 (0.09)	−0.11 (−0.39, 0.16)	0.06
	VDI	−0.19 (0.10)	0.27 (−0.05, 0.60)	−0.21 (0.06)	−0.20 (−0.44, 0.04)	
	VDS	−0.46 (0.13)	**Ref**	−0.01 (0.10)	Ref	
**Developmental Social Disorder**	VDD	−0.18 (0.07)	0.25 (−0.09, 0.58)	−0.14 (0.08)	0.04 (−0.22, 0.29)	0.52
	VDI	−0.25 (0.08)	0.18 (−0.16, 0.52)	−0.24 (0.06)	−0.05 (−0.29, 0.18)	
	VDS	−0.43 (0.15)	Ref	−0.18 (0.10)	Ref	
**Emotional Behavioral Disorder**	VDD	−0.01 (0.08)	0.38 (0.05, 0.70)	−0.14 (0.09)	−0.19 (−0.49, 0.11)	0.05
	VDI	−0.07 (0.10)	0.31 (−0.04, 0.66)	−0.14 (0.06)	−0.19 (−0.46, 0.08)	
	VDS	−0.39 (0.15)	Ref	0.05 (0.12)	Ref	
**Resiliency**	VDD	0.15 (0.06)	−0.13 (−0.43, 0.17)	0.13 (0.10)	−0.02 (−0.30, 0.26)	0.86
	VDI	0.22 (0.07)	−0.06 (−0.37, 0.25)	0.18 (0.07)	0.04 (−0.21, 0.28)	
	VDS	0.28 (0.14)	Ref	0.15 (0.10)	Ref	
**Functional Impairment**	VDD	−0.16 (0.07)	0.31 (−0.01, 0.64)	−0.12 (0.09)	−0.15 (−0.43, 0.13)	0.10
	VDI	−0.21 (0.09)	0.27 (−0.07, 0.62)	−0.14 (0.07)	−0.16 (−0.43, 0.10)	
	VDS	−0.48 (0.15)	Ref	0.03 (0.11)	Ref	

* Regression model adjusted for HIV status, developmental stage, and time.

**Table 4:** Vitamin D in relation to developmental disorder probability indices among pre-adolescent Ugandan children.

		Model 1	Model 2	Model 3
	VD Groups	SMD (95% CI)	SMD (95% CI)	SMD (95% CI)
**Autism Spectrum Disorder**	VDD	0.34 (0.02, 0.66)	0.32 (0.00, 0.64)	0.32 (0.01, 0.63)
	VDI	**0.21 (−0.11, 0.52)**	**0.11 (−0.20, 0.42)**	**0.14 (−0.18, 0.45)**
	VDS	**Ref**	**Ref**	**Ref**
**Attention Deficit and Hyperactivity Disorder**	VDD	0.34 (0.03, 0.63)	0.38 (0.07, 0.70)	0.46 (0.15, 0.78)
	VDI	**0.27 (−0.05, 0.60)**	**0.26 (−0.07, 0.59)**	**0.32 (−0.01, 0.65)**
	VDS	**Ref**	**Ref**	**Ref**
**Developmental Social Disorder**	VDD	0.25 (−0.09, 0.58)	0.26 (−0.10, 0.60)	0.28 (−0.06, 0.61)
	VDI	0.18 (−0.15, 0.52)	0.19 (−0.17, 0.55)	0.21 (−0.15, 0.56)
	VDS	Ref	Ref	Ref
**Emotional Behavioral Disorder**	VDD	0.38 (0.05, 0.70)	0.40 (0.05, 0.74)	0.45 (0.11, 0.80)
	VDI	**0.31 (−0.04, 0.66)**	**0.24 (−0.12, 0.60)**	**0.30 (−0.04, 0.65)**
	VDS	**Ref**	**Ref**	**Ref**
**Resiliency**	VDD	−0.13 (−0.43, 0.17)	−0.20 (−0.48, 0.10)	−0.18 (−0.48, 0.11)
	VDI	−0.06 (−0.37, 0.25)	−0.14 (−0.45, 0.17)	−0.12 (−0.43, 0.19)
	VDS	Ref	Ref	Ref
**Functional Impairment**	VDD	0.31 (−0.01, 0.64)	0.35 (0.02, 0.68)	0.38 (0.05, 0.71)
	VDI	0.27 (−0.07, 0.62)	**0.24 (−0.11, 0.58)**	**0.27 (−0.07, 0.61)**
	VDS	Ref	**Ref**	**Ref**

Note: Multivariable regression models adjusted for HIV status and time (Model 1), caregiver (education, age, anxiety, sex, social support, social standing, recent life stress, adverse lived experiences, and depression (Model 2), and (caregiver factors in model 2+ child Apgar scores and birth weight [Model 3]). The bolded numbers are statistically significant.

## Data Availability

The data presented in this study is available on request from the corresponding author but subject to data sharing agreements.

## Appendix A

**Table A1:** Mean and Standard Deviation of probability scores for six developmental disorders overall and according to perinatal HIV status.

Outcomes	Baseline (*n* = 605)	Month 6 (*n* = 597)	Month 12 (*n* = 444)	*p*-Trend
	Mean (SD)	Mean (SD)	Mean (SD)	
**Regardless of HIV Status**				
Autism Spectrum Disorder	−0.00 (0.98)	−0.15 (0.97)	−0.24 (0.93)	<0.0001
Attention Deficit Hyperactivity Disorder	−0.04 (1.03)	−0.17 (1.00)	−0.26 (1.03)	0.0001
Developmental Social Disorder	−0.06 (0.98)	−0.24 (0.95)	−0.33 (0.89)	<0.001
Emotional Behavior Disorder	0.02 (0.97)	−0.09 (1.03)	−0.15 (0.99)	0.03
Resiliency	0.07 (1.01)	0.22 (0.94)	0.23 (0.90)	<0.0001
Functional Impairment Index	−0.01 (1.01)	−0.15 (1.05)	-0.31 (0.96)	<0.0001
**CPHIV**	(***n* = 199**)	(***n* = 198**)	(***n* = 136**)	
	**Mean** (**SD**)	**Mean** (**SD**)	**Mean** (**SD**)	
Autism Spectrum Disorder	−0.04 (0.96)	−0.18 (0.90)	−0.29 (0.88)	0.01
Attention Deficit Hyperactivity Disorder	−0.11 (1.04)	−0.26 (0.92)	−0.29 (0.94)	0.17
Developmental Social Disorder	−0.12 (0.98)	−0.30 (0.86)	−0.36 (0.77)	0.003
Emotional Behavior Disorder	−0.06 (0.97)	−0.13 (0.92)	−0.21 (0.94)	0.25
Resiliency Index	0.14 (1.04)	0.21 (0.95)	0.19 (0.76)	0.20
Functional Impairment Index	−0.01 (1.07)	−0.14 (0.99)	−0.27 (0.93)	0.02
**CHEU**	(***n* = 196**)	(***n* = 194**)	(***n* = 161**)	
	**Mean** (**SD**)	**Mean** (**SD**)	**Mean** (**SD**)	
Autism Spectrum Disorder	0.05 (0.92)	−0.10 (0.99)	−0.20 (0.94)	0.02
Attention Deficit Hyperactivity Disorder	−0.02 (0.99)	−0.10 (1.00)	−0.28 (1.03)	0.02
Developmental Social Disorder	−0.04 (0.93)	−0.18 (0.93)	−0.32 (0.93)	0.003
Emotional Behavior Disorder	0.01 (0.96)	−0.03 (1.06)	−0.12 (0.98)	0.39
Resiliency Index	0.05 (0.94)	0.24 (0.94)	0.26 (0.94)	0.03
Functional Impairment Index	0.03 (0.97)	−0.10 (1.09)	−0.27 (0.95)	0.006
**CHUU**	(***n* = 201**)	(***n* = 201**)	(***n* = 147**)	
	**Mean** (**SD**)	**Mean** (**SD**)	**Mean** (**SD**)	
Autism Spectrum Disorder	−0.02 (1.05)	−0.18 (1.03)	−0.25 (0.97)	0.02
Attention Deficit and Hyperactivity Disorder	−0.01 (1.05)	−0.16 (1.08)	−0.23 (1.11)	0.04
Developmental Social Disorder	−0.03 (1.03)	−0.24 (1.06)	−0.30 (0.99)	0.004
Emotional Behavior Disorder	−0.04 (0.99)	−0.11 (1.10)	−0.14 (1.02)	0.31
Resiliency Index	0.03 (1.06)	0.24 (0.93)	0.23 (0.98)	0.02
Functional Impairment Index	−0.06 (1.02)	−0.22 (1.09)	−0.29 (1.01)	0.05

## Appendix B

**Table A2:** Relationship of select child and caregiver non-nutritional variables to developmental disorders, resiliency, and functioning in Ugandan children.

	Autism Spectrum Disorder	Attention Deficit and Hyperactivity Disorder	Emotional Behavioral Disorder	Developmental Social Disorder	Resiliency	Functional Impairment
	SMD (95% CI)	SMD (95% CI)	SMD (95% CI)	SMD (95% CI)	SMD (95% CI)	SMD (95% CI)
**Childbirth Weight**						
	−0.14 (−0.29, 0.01)	**−0.19** (**−0.34, −0.03**)	0.01 (−0.16, 0.17)	−0.09 (−0.24, 0.06)	0.16 (−0.01, 0.32)	−0.10 (−0.25, 0.05)
**Child Apgar Score**						
	**−0.11** (**−0.19, −0.04**)	−0.03 (−0.11, 0.04)	−0.06 (−0.14, 0.01)	**−0.11** (**−0.19, −0.03**)	**0.11**(**0.03, 0.18**)	**−0.13** (**−0.21, −0.05**)
**Caregiver Sex**						
	0.02 (−0.22, 0.26)	−0.12 (−0.36, 0.12)	−0.12 (−0.35, 0.11)	−0.03 (−0.27, 0.21)	−0.04 (−0.29, 0.21)	−0.10 (−0.34, 0.14)
**Caregiver Age** (**years**)						
20–30	0.15 (−0.04, 0.35)	0.14 (−0.06, 0.33)	0.25 (0.05, 0.44)	0.12 (−0.07, 0.31)	−0.02 (−0.21, 0.17)	0.21 (0.01, 0.42)
31−40	−0.03 (−0.21, 0.14)	0.07 (−0.11, 0.25)	**0.05 (−0.12, 0.22)**	0.04 (−0.13, 0.22)	−0.01 (−0.19, 0.17)	**0.03 (−0.16, 0.21)**
41–74	Ref	Ref	**Ref**	Ref	Ref	**Ref**
**Caregiver Adverse Life Events**						
0	−0.53 (−0.73, −0.32)	−0.49 (−0.70, −0.27)	−0.67 (−0.90, −0.45)	−0.52 (−0.73, −0.31)	0.19 (−0.03, 0.40)	−0.66 (−0.88, −0.43)
1	**−0.34 (−0.53, −0.16)**	**−0.35 (−0.55, −0.14)**	**−0.43 (−0.66, −0.21)**	**−0.25 (−0.44, −0.05)**	−0.08 (−0.28, 0.11)	**−0.39 (−0.60, −0.19)**
2	**−0.23 (−0.43, −0.04)**	**−0.19 (−0.40, 0.02)**	**−0.24 (−0.45, −0.03)**	**−0.14 (−0.32, 0.03)**	−0.02 (−0.20, 0.15)	**−0.21 (−0.41, −0.01)**
3+	**Ref**	**Ref**	**Ref**	**Ref**	Ref	**Ref**
**Caregiver Recent Life Stress Score**						
0	−0.40 (−0.60, −0.20)	−0.31 (−0.52, −0.11)	−0.45 (−0.67, −0.23)	**−0.39 (−0.58, −0.20)**	0.12 (−0.07, 0.32)	−0.54 (−0.78, −0.31)
1	**−0.30 (−0.48, −0.12)**	**−0.26 (−0.43, −0.08)**	**−0.47 (−0.66, −0.28)**	**−0.26 (−0.43, −0.09)**	0.04 (−0.13, 0.22)	**−0.38 (−0.59, −0.18)**
2	**−0.22 (−0.42, −0.02)**	**−0.12 (−0.31, 0.06)**	**−0.24 (−0.46, −0.02)**	**−0.08 (−0.27, 0.10)**	−0.03(−0.21, 0.15)	**−0.21 (−0.44, 0.03)**
3+	**Ref**	**Ref**	**Ref**	**Ref**	Ref	**Ref**
**Caregiver Depression**						
0	**−0.25 (−0.43, −0.06)**	−0.26 (−0.46, −0.07)	−0.52 (−0.71, −0.33)	**−0.27 (−0.45, −0.09)**	−0.02(−0.21, 0.16)	−0.40 (−0.60, −0.21)
1	**−0.04 (−0.21, 0.41)**	**−0.05 (−0.23, 0.13)**	**−0.24 (−0.43, −0.06)**	**−0.01 (−0.17, 0.15)**	−0.11(−0.28, 0.06)	**−0.11 (−0.29, 0.07)**
2	**Ref**	**Ref**	**Ref**	**Ref**	Ref	**Ref**
**Caregiver Anxiety**						
0	**−0.33 (−0.61, −0.05)**	−0.44 (−0.71, −0.17)	−0.70 (−0.96, −0.41)	**−0.36 (−0.65, −0.07)**	0.03 (−0.23, 0.29)	−0.64 (−0.93, −0.35)
1	**−0.23 (−0.44, 0.00)**	**−0.27 (−0.49, −0.05)**	**−0.53 (−0.75, −0.31)**	**−0.25 (−0.45, −0.04)**	−0.11(−0.32, 0.10)	**−0.34 (−0.56, −0.12)**
2	**−0.13 (−0.34, 0.08)**	**−0.19 (−0.40, 0.03)**	**−0.37 (−0.58, −0.16)**	**−0.13 (−0.32, 0.05)**	−0.16(−0.35, 0.03)	**−0.17 (−0.37, 0.04)**
3+	**Ref**	**Ref**	**Ref**	**Ref**	Ref	**Ref**
**Caregiver Social Support**						
0	0.13 (−0.11, 0.37)	−0.03 (−0.27, 0.21)	0.04 (−0.19, 0.27)	−0.02 (−0.25, 0.20)	0.17 (−0.05, 0.39)	0.02 (−0.22, 0.25)
1	−0.00 (−0.22, 0.22)	0.05 (−0.18, 0.28)	0.02 (−0.20, 0.24)	−0.08 (−0.30, 0.13)	0.14 (−0.09, 0.36)	−0.04 (−0.27, 0.19)
2	0.19 (0.01, 0.36)	0.13 (−0.06, 0.33)	**0.26 (0.06, 0.46)**	0.17 (−0.00, 0.35)	−0.04 (−0.22, 0.14)	0.24 (0.05, 0.44)
3+	Ref	Ref	Ref	Ref	Ref	Ref

Note: The bolded numbers are statistically significant.

## References

[1] Jia F., Shan L., Wang B., Li H., Miao C., Xu Z., Lin C.-P., Saad K. (2018). Bench to BEDSIDE REVIEW: Possible role of vitamin D in autism spectrum disorder. Psychiatry Res..

[2] Lee B.K., Eyles D.W., Magnusson C., Newschaffer C.J., McGrath J.J., Kvaskoff D., Ko P., Dalman C., Karlsson H., Gardner R.M. (2019). Developmental vitamin D and autism spectrum disorders: Findings from the Stockholm Youth Cohort. Mol. Psychiatry.

[3] Hemal A., Garg S., Agarwal A., Singh M. (2016). Vitamin D deficiency in HIV infected children. Eur. J. Pediatr..

[4] Mirza A., Wells S., Gayton T., Smotherman C., Rathore A., Kraemer D., Rathore M. (2016). Vitamin D Deficiency in HIV-Infected Children. South. Med. J..

[5] Klassen K.M., Fairley C.K., Kimlin M.G., Hocking J., Kelsall L., Ebeling P.R. (2016). Vitamin D Deficiency is Common in HIV-Infected Southern Australian Adults. Antivir. Ther..

[6] Mansueto P., Seidita A., Vitale G., Gangemi S., Iaria C., Cascio A. (2015). Vitamin D Deficiency in HIV Infection: Not only a Bone Disorder. BioMed Res. Int..

[7] Gedela K., Edwards S.G., Benn P., Grant A.D. (2014). Prevalence of vitamin D deficiency in HIV-positive, antiretroviral treatment-naive patients in a single centre study. Int. J. STD AIDS.

[8] Orkin C., Wohl D.A., Williams A., Deckx H. (2014). Vitamin D Deficiency in HIV: A shadow on long-term management?. AIDS Rev..

[9] Chokephaibulkit K., Saksawad R., Bunupuradah T., Rungmaitree S., Phongsamart W., Lapphra K., Maleesatharn A., Puthanakit T. (2013). Prevalence of vitamin D deficiency among perinatally HIV-infected thai adolescents receiving antiretroviral therapy. Pediatr. Infect. Dis. J..

[10] Meyzer C., Frange P., Chappuy H., Desse B., Veber F., Le Clesiau H., Friedlander G., Blanche S., Souberbielle J.C., Tréluyer J.M. (2013). Vitamin D Deficiency and Insufficiency in HIV-infected children and young adults. Pediatr. Infect. Dis. J..

[11] Pinzone M.R., Di Rosa M., Malaguarnera M., Madeddu G., Foca E., Ceccarelli G., d’Ettorre G., Vullo V., Fisichella R., Cacopardo B. (2013). Vitamin D deficiency in HIV infection: An underestimated and undertreated epidemic. Eur. Rev. Med. Pharmacol. Sci..

[12] Prime K., Pelham E.R. (2013). High rates of vitamin D deficiency in a London adolescent HIV clinic. HIV Med..

[13] Van Den Bout-Van Den Beukel C.J.P., Fievez L., Michels M., Sweep F.C.G.J., Hermus A.R.M.M., Bosch M.E.W., Burger D.M., Bravenboer B., Koopmans P.P., Van Der Ven A.J. (2008). Vitamin D Deficiency among HIV Type 1-Infected Individuals in the Netherlands: Effects of Antiretroviral Therapy. AIDS Res. Hum. Retrov..

[14] Giusti A., Penco G., Pioli G. (2011). Vitamin D deficiency in HIV-infected patients: A systematic review. Nutr. Diet. Suppl..

[15] Jao J., Freimanis L., Mussi-Pinhata M.M., Cohen R.A., Monteiro J.P., Cruz M.L., Branch A., Sperling R.S., Siberry G.K., NISDI LILAC Protocol (2017). Severe Vitamin D Deficiency in Human Immunodeficiency Virus-Infected Pregnant Women is Associated with Preterm Birth. Am. J. Perinatol..

[16] Holick M.F. (2007). Vitamin D Deficiency. N. Engl. J. Med..

[17] Holick M.F. (2010). Vitamin D: Extraskeletal health. Endocrinol. Metab. Clin. N. Am..

[18] Casey C.F., Slawson D.C., Neal L.R. (2010). VItamin D supplementation in infants, children, and adolescents. Am. Fam. Physician.

[19] Brewer L.D., Thibault V., Chen K.-C., Langub M.C., Landfield P.W., Porter N.M. (2001). Vitamin D Hormone Confers Neuroprotection in Parallel with Downregulation of L-Type Calcium Channel Expression in Hippocampal Neurons. J. Neurosci..

[20] Cui X., Pelekanos M., Liu P.-Y., Burne T.H.J., McGrath J.J., Eyles D.W. (2013). The vitamin D receptor in dopamine neurons; its presence in human substantia nigra and its ontogenesis in rat midbrain. Neuroscience.

[21] Eyles D.W., Smith S., Kinobe R., Hewison M., McGrath J.J. (2005). Distribution of the Vitamin D receptor and 1α-hydroxylase in human brain. J. Chem. Neuroanat..

[22] Eyles D.W., Liu P.Y., Josh P., Cui X. (2014). Intracellular distribution of the vitamin D receptor in the brain: Comparison with classic target tissues and redistribution with development. Neuroscience.

[23] Rajmohan V., Mohandas E. (2007). The limbic system. Indian J. Psychiatry.

[24] Zong L., Chu P., Huang P., Guo Y., Lv Y. (2017). Effect of vitamin D on the learning and memory ability of FGR rat and NMDA receptor expression in hippocampus. Exp. Ther. Med..

[25] Lardner A.L. (2015). Vitamin D and hippocampal development-the story so far. Front. Mol. Neurosci..

[26] Alfieri D.F., Lehmann M.F., Oliveira S.R., Flauzino T., Delongui F., de Araújo M.C.M., Simão A.N., Reiche E.M. (2017). Vitamin D deficiency is associated with acute ischemic stroke, C-reactive protein, and short-term outcome. Metab. Brain Dis..

[27] Groves N.J., McGrath J.J., Burne T.H. (2014). Vitamin D as a Neurosteroid Affecting the Developing and Adult Brain. Annu. Rev. Nutr..

[28] Wang Z., Ding R., Wang J. (2020). The Association between Vitamin D Status and Autism Spectrum Disorder (ASD): A Systematic Review and Meta-Analysis. Nutrients.

[29] Gong Z.-L., Luo C.-M., Wang L., Shen L., Wei F., Tong R.-J., Liu Y. (2014). Serum 25-hydroxyvitamin D levels in Chinese children with autism spectrum disorders. Neuroreport.

[30] Göksügür S.B., Tufan A.E., Semiz M., Gunes C., Bekdaş M., Tosun M., Demircioglu F. (2014). Vitamin D status in children with attention-deficit-hyperactivity disorder. Pediatr. Int..

[31] Kamal M., Bener A., Ehlayel M.S. (2014). Is high prevalence of vitamin D deficiency a correlate for attention deficit hyperactivity disorder?. ADHD Atten. Deficit Hyperact. Disord..

[32] Kotsi E., Kotsi E., Perrea D.N. (2019). Vitamin D levels in children and adolescents with attention-deficit hyperactivity disorder (ADHD): A meta-analysis. ADHD Atten. Deficit Hyperact. Disord..

[33] Bener A., Kamal M. (2014). Predict attention deficit hyperactivity disorder? Evidence-based medicine. Glob. J. Health Sci..

[34] Yakah W., Fenton J.I., Sikorskii A., Zalwango S.K., Tuke R., Musoke P., Boivin M.J., Giordani B., Ezeamama A.E. (2019). Serum Vitamin D is Differentially Associated with Socioemotional Adjustment in Early School-Aged Ugandan Children According to Perinatal HIV Status and in Utero/Peripartum Antiretroviral Exposure History. Nutrients.

[35] Husmann C., Frank M., Schmidt B., Jöckel K.H., Antel J., Reissner V., Libuda L., Hebebrand J., Föcker M. (2017). Low 25(OH)-vitamin D concentrations are associated with emotional and behavioral problems in German children and adolescents. PLoS ONE.

[36] Hansen A.L., Ambroziak G., Thornton D., Mundt J.C., Kahn R.E., Dahl L., Waage L., Kattenbraker D., Araujo P., Murison R. (2020). Vitamin D Supplementation during Winter: Effects on Stress Resilience in a Randomized Control Trial. Nutrients.

[37] Terock J., Hannemann A., Janowitz D., Müller J., Völzke H., Grabe H.J. (2020). Vitamin D levels are associated with trait resilience but not depression in a general population sample. Brain Behav..

[38] Zablotsky B., Black L.I., Maenner M.J., Schieve L.A., Blumberg S.J. (2015). Estimated prevalence of autism and other developmental disabilities following questionnaire changes in the 2014 National Health Interview Survey. Natl. Health Stat. Rep..

[39] Sharma S.R., Gonda X., Tarazi F.I. (2018). Autism Spectrum Disorder: Classification, diagnosis and therapy. Pharmacol. Ther..

[40] Lake J.E., Adams J.S. (2011). Vitamin D in HIV-Infected Patients. Curr. HIV AIDS Rep..

[41] Awadu J.E., Sikorskii A., Zalwango S., Coventry A., Giordani B., Ezeamama A.E. (2022). Developmental Disorder Probability Scores at 6–18 Years Old in Relation to In-Utero/Peripartum Antiretroviral Drug Exposure among Ugandan Children. Int. J. Environ. Res. Public Health.

[42] Reynolds C., Kamphaus R. (2015). Behavior Assessment System for Children—Third Edition (BASC-3).

[43] Zhou X., Reynolds C., Kamphaus R.W. (2022). Diagnostic utility of Behavior Assessment System for Children-3 for children and adolescents with autism. Appl. Neuropsychol. Child..

[44] Faraone S.V., Banaschewski T., Coghill D., Zheng Y., Biederman J., Bellgrove M.A., Newcorn J.H., Gignac M., Al Saud N.M., Manor I. (2021). The World Federation of ADHD International Consensus Statement: 208 Evidence-based conclusions about the disorder. Neurosci. Biobehav. Rev..

[45] Bener A., Khattab A.O., Al-Dabbagh M.M. (2014). Is high prevalence of Vitamin D deficiency evidence for autism disorder?: In a highly endogamous population. J. Pediatr. Neurosci..

[46] Zhang X., Wang L., Han J., Xie L., Wu N., Nyirenda R., Shumba K., Simwanza W., Kulyk O., Chilapondwa V. (2021). In utero antiretroviral exposure and sociodemographic characteristics on neurodevelopment of HIV-exposed uninfected children versus HIV-unexposed uninfected healthy children in Malawi. Int. J. Gynecol. Obstet..

[47] Grant W.B., Wimalawansa S.J., Holick M.F., Cannell J.J., Pludowski P., Lappe J.M., Pittaway M., May P. (2015). Emphasizing the Health Benefits of Vitamin D for Those with Neurodevelopmental Disorders and Intellectual Disabilities. Nutrients.

[48] Indika N.R., Frye R.E., Rossignol D.A., Owens S.C., Senarathne U.D., Grabrucker A.M., Perera R., Engelen M.P.K.J., Deutz N.E.P. (2023). The Rationale for Vitamin, Mineral, and Cofactor Treatment in the Precision Medical Care of Autism Spectrum Disorder. J. Pers. Med..

[49] Kerley C.P., Power C., Gallagher L., Coghlan D. (2017). Lack of effect of vitamin D(3) supplementation in autism: A 20-week, placebo-controlled RCT. Arch. Dis. Child..

[50] Lai M.-C., Lombardo M.V., Chakrabarti B., Ruigrok A.N., Bullmore E.T., Suckling J., Auyeung B., Happé F., Szatmari P., Baron-Cohen S. (2018). Neural self-representation in autistic women and association with ‘compensatory camouflaging’. Autism.

[51] Lord C., Risi S., Lambrecht L., Cook E.H., Leventhal B.L., DiLavore  P.C., Pickles A., Rutter M. (2000). The Autism Diagnostic Observation Schedule—Generic: A standard measure of social and communication deficits associated with the spectrum of autism. J. Autism Dev. Disord..

[52] Lord C., Rutter M., Le Couteur A. (1994). Autism Diagnostic Interview-Revised: A revised version of a diagnostic interview for caregivers of individuals with possible pervasive developmental disorders. J. Autism Dev. Disord..

